# Anticancer Activity of Essential Oils and Other Extracts from Aromatic Plants Grown in Greece

**DOI:** 10.3390/antiox8080290

**Published:** 2019-08-07

**Authors:** Eleni Fitsiou, Aglaia Pappa

**Affiliations:** 1 Department of Molecular Biology and Genetics, Democritus University of Thrace, University Campus, Dragana, 68100 Alexandroupolis, Greece; 2 European Research Institute for the Biology of Ageing, University Medical Center Groningen, University of Groningen, Antonius Deusinglaan 1, 9713 AV Groningen, The Netherlands

**Keywords:** aromatic plants, natural extracts, Greece, antiproliferative activity, cancer

## Abstract

Aromatic plants have a long and significant history in the traditional medicine of many countries. Nowadays, there is an increasing interest in investigating the biological properties of aromatic plant extracts mainly due to their diversity, high availability, and low toxicity. Greece is abundant in aromatic plants, which can be attributed to the country’s geographical position, the morphology of its landscape, and its numerous mountainous and insular areas. In the past 15 years, a number of aromatic plant extracts of Greek origin have been studied for their bioactivities, including their antiproliferative potential against different types of cancer. Although the pharmacological activities of specific species of Greek origin have been reviewed before, no gathered information on explicitly Greek species exist. In this review, we summarize existing data on the antiproliferative activity of extracts isolated from Greek aromatic plants and discuss their molecular mode(s) of action, where available, in order to identify promising extracts for future research and link chemical constituents responsible for their activity. We conclude that essentials oils are the most frequently studied plant extracts exhibiting high diversity in their composition and anticancer potential, but also other extracts appear to be worthy of further investigation for cancer chemoprevention.

## 1. Introduction

Aromatic plants take their name from the distinct aroma they produce, which is attributed to a mixture of volatile compounds extracted as essential oils. They constitute 10% of the plant kingdom (17,000 species) and are distributed all over the world, mostly in temperate and warm countries [1]. Greece is abundant in aromatic plants. Its geographical position, the morphology of its landscape, and the numerous mountainous and insular areas (109 out of 225 habitat types encountered in the European Union are found in Greece) [2] contribute to the country’s high plant diversity and endemism (6600 species and subspecies) [3], explaining its long-documented ethnopharmacological history. Most of the plants collected for research purposes in the Greek area have been obtained from Central Greece and Crete and belong to the Lamiaceae family [4]. In recent years, increased interest has been focused on studying the biological properties of aromatic plant extracts mostly due to their high availability and few side-effects. Among their reported properties is their ability to affect proliferation of cancer cells and induce apoptosis, one of the most important features of cancer chemoprevention.

In Greece, cancer is the second leading cause of death after cardiovascular diseases. With a population of approximately 11,000,000, there was an expected number of 67,400 new cancer cases and 33,200 deaths in 2018 (~6% and 3% of the population, respectively) [5]. Although overall cancer rates have not increased since 2014 (~29,000 deaths), there has been an increase in the number of specific cancer types including lung cancer in men, colorectal, breast, pancreatic, and prostate cancer [6]. According to the International Agency for Research on Cancer (IARC) and the World Cancer Observatory, Greece was in the 28th place in cancer rates globally for men and women in 2018 with lung cancer having the highest rates in incidence and mortality for both sexes (one of the highest rates for men in Europe) followed by breast and colorectal cancer [5,7]. Unfortunately, there are no data available on the correlation between the anticancer activity of aromatic plants and tumor incidence in Greece. It is challenging, though, to investigate how isolated food components may contribute to cancer incidence and/or affect mortality rates as assessing diet through dietary patterns has become popular in diet–disease investigations [8]. However, cancer reports suggest that foods rich in vitamins and phytochemicals can act as chemopreventive agents reducing the risk of various types of cancer, especially colorectal cancer [9,10]. In addition, there are studies with Greek participants that show that the adoption of the Mediterranean diet (characterized by high intake of fruit, vegetables, nuts, whole grains, and olive oil; a moderate intake of fish and dairy products; and low intake of meat, especially red meat), reduce cancer-related and total mortality [11,12].

Currently, literature is lacking thorough research on the anticancer activity of aromatic extracts of Greek origin, which would be very useful for the appraisal and comparison of the data in this increasingly growing field. Therefore, the present review summarizes all the existing data on various plant extracts discussing their molecular mode(s) of action where available.

## 2. Methods

We performed a systematic search on Pubmed, Google scholar, and Scopus databases using the keywords “aromatic plants”, “plant extracts”, “cancer”, “antiproliferative”, “anticancer”, “Greece”, “Greek”. We focused our research on selected studies dealing only with the activity of aromatic plant extracts and not plants in general or isolated compounds against cancer in vitro and/or in vivo. We also excluded any study were the plant material was not harvested from the Greek area.

## 3. Results

**Figure 1:**
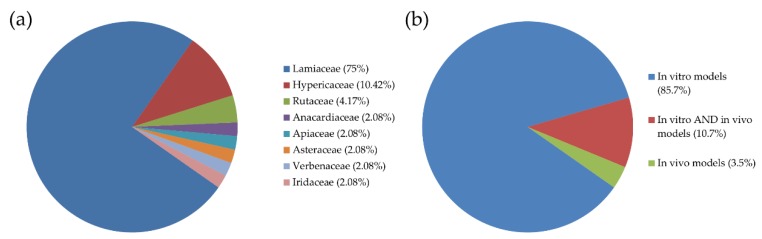
(**a**) The percentage of plant families of the species studied for their antiproliferative activity. (**b**) The percentage of studies on aromatic plant extracts performed in (i) in vitro models, (ii) in vitro and in vivo models, or (iii) only in in vivo models.

Our research was restricted only to aromatic plant extracts and not plants in general or isolated compounds. We found 28 research studies conducted from 1996 to July 2019, dealing mostly with essential oils and methanolic extracts. In these studies, 48 species were investigated for their properties, the majority of which belong to the Lamiaceae family, in accordance with previous published data [4] (Figure 1a). Only four studies included in vivo models; three of them were investigating *Pistacia lentiscus* var. *Chia* extracts and one the essential oil of *Origanum Onites* L. (Figure 1b). In the majority of these studies, human cancer cells were used to evaluate cytotoxicity, with colon cancer cells being the most frequently used, followed by hepatocellular and breast carcinomas. Finally, only four studies included testing the cytotoxicity on normal cells, too (PBMCs, African green monkey kidney, rabbit skin, mouse adipose areolar, and HaCat cells). The available data are summarized in Table 1.

*Pistacia lentiscus* var. *Chia,* commonly known as Chios mastic, is the plant mostly studied (nine studies) and is the only one with available and more detailed information on its molecular mode(s) of action on various cancer models. *Pistacia lentiscus* var. *Chia* is a variety endemic to Chios island, with high economic value as it is the only variety that produces resin when its bark is scored. When the essential oil of the resin was incubated with breast, colon, and ovarian adenocarcinoma cell lines (MCF-7, LoVo, 2008) for 3 h, followed by a 21 h recovery period, it demonstrated the weakest activity compared to other *Pistacia* species originating from other countries (EC_50_ > 500 μg/mL) [13]. The essential oil has been also studied against human leukemic and melanoma cells and mouse lung carcinoma (K562, Lewis lung carcinoma cells, B16, respectively) and also in vivo in a lung cancer mouse model. These studies revealed that mastic oil reduces cell viability and tumor growth. In addition, it was shown that it induces apoptosis in cancer cells, reduces the expression of chemokines, limits their invasive and adhesive potential, and is an inhibitor of angiogenesis via the reduction of VEGF expression and neovascularisation [14,15,16,17]. When the oil was tested against colon carcinoma, it showed significant dose- and time-dependent cytotoxicity in vitro and tumor reducing capacity in vivo, which was higher than the activity of its major components, α-pinene and myrcene, tested either alone or in combination in the ratio found in the oil mixture. Furthermore, downregulation in the levels of the proliferation markers Ki-67 and survivin and attenuation of cancer cell migration was observed [18]. A hexane and an ethanol extract of mastic have been also studied for their anticancer potential in vitro against a human colon cancer cell line (HCT116) and in vivo in an experimental colon cancer mouse model. Both extracts caused dose-dependent inhibition of cell growth, while the hexane extract also caused tumor growth suppression. Furthermore, the extracts caused G1 cell cycle arrest, induction of apoptosis via the activation of caspases 3, 8 and 9, while it was found that the cell death caused by the ethanol extract is independent of p53, Bcl-2, and FADD [19,20,21].

**Table 1:** Summary of the antiproliferative activity of extracts isolated from Greek aromatic plants. The cell lines are human unless otherwise stated. N.d. = not determined. (−) in major component and collection location columns indicates that the authors report no data on the major components and collection location, respectively. (--) in dose/concentration column indicates that the concentrations used were not reported.

Family	Species	Extract	Major Components	Collection Location	Type of Cell	Dose/ Concentration	Effect	Ref.
Anacard-iaceae	*Pistacia lentiscus* var*. Chia*	Essential oil	α-Pinene (67.71%) Myrcene (18.81%)	Commercial	Colon carcinomas Caco2 and HT-29 Mouse colon carcinoma CT-26	0.000445–0.89 mg/mL	Caco2 EC_50_ 48 h = 0.0368 ± 0.0225 mg/mL EC_50_ 72 h = 0.0176 ± 0.0035 mg/mL HΤ-29 ΕC_50_ 48 h = 0.1751 ± 0.0028 mg/mL ΕC_50_ 72 h = 0.0762 ± 0.0057 mg/mL CT26 EC_50_ 48 h = 0.1335 ± 0.054 mg/mL ΕC_50_ 72 h = 0.0104 ± 0.0004 mg/mL	[18]
					Colon cancer mouse model (in vivo)	0.58 g/kg b.w.	44–52% tumor volume inhibition Cancer cell migration attenuation Downregulation in the expression levels of Ki-67 and survivin	
		Essential oil	α-Pinene (72.93%) Myrcene (13.57%)	Commercial	Ovarian adenocarcinoma 2008 breast adenocarcinoma MCF-7 colon carcinoma LoVo	--	EC_50_ > 500 μg/mL	[13]
		Essential oil	−	Commercial	chronic myelogenous leukemia K562 Mouse B16 melanoma	0.01–0.1% *v*/*v*	K562: Reduction of viability. Induction of apoptosis; Reduction of VEGF release levels; Inhibition of endothelial cells proliferation and neovascularization; Erk1/2 reduction B16: Reduction of VEGF release levels	[14]
		Essential oil	α-Pinene (~70%)	Commercial	Mouse Lewis lung carcinoma	0.01–0.02% *v*/*v*	Cell proliferation suppression; VEGF release reduction; Chemokine release reduction; GTPases Ras, RhoA and NF-*κ*B-dependent reporter gene expression decrease	[16]
					Lung cancer mouse model (in vivo)	45 mg/Kg b.w.	Tumor growth suppression; Apoptosis induction; Neovascularization reduction; Chemokine expression reduction GTPases, Ras, RhoA and NF-*κ*B-dependent reporter gene expression decrease	
		Essential oil	−	−	Mouse Lewis lung carcinoma	0.01% *v*/*v*	PTEN, E2F7, HMOX1 increase; NOD1 decrease; Apoptosis induction; Inflammation decrease	[17]
		Essential oil	−	Commercial	Mouse Lewis lung carcinoma	0.01–0.04% *v*/*v*	Reduction of MMP-2, ICAM-1 and VCAM-1 expression levels; attenuation of f-actin fiber formation; limitation of cell invasiveness; impairment of tumor cell adhesive interactions and neovascularization potential	[15]
		Mastic gum hexane extract	Caryophyllene	−	Colon carcinoma HCT116	25–100 µg/mL	Dose-dependent growth inhibition G1 cell cycle arrest Induction of apoptosis Activation of caspases 3, 8, 9 PARP degradation	[20]
		Mastic gum hexane extract	−	−	Colon cancer mouse model (in vivo)	200 mg/Kg b.w.	Tumor growth suppression	[21]
		Mastic gum ethanol extract	−	−	Colon carcinoma HCT116	0.2–0.6% *v*/*v*	Dose-dependent inhibition of cell growth G1 cell cycle arrest cell death is independent of p53, Bcl-2 and FADD Induction of apoptosis Activation of caspases 3, 8, 9 PARP degradation	[19]
Iridaceae	*Crocus sativus* L.	Ethanolic extract	trans-crocin 1 (56.4%) trans-crocin 2 (19.2%) cis-crocin 1 (6.8%)	Commercial (Association of Crocus Producers)	Rat glioma C6	0.5–10 mg/mL	EC_50_ = 3 mg/mL No apoptosis induction Colony formation impairment Calpain-dependent cell death, possibly autophagy Synergistic effect with temozolomide at certain combinations	[22]
Verbena-ceae	*Lippia citriodora*	Essential oil	Neral (*cis*-citral) (17.2%) Geranial (*trans*-citral) (26.4%)	Athens	Hepatocellular carcinoma HepG2 Breast adenocarcinoma MCF-7 Colon adenocarcinoma Caco2 Leukemic monocytes THP-1 Malignant melanoma A375	0.64–920 µg/mL	HepG2 EC_50_ = 74 ± 2.8 μg/mL MCF-7 EC_50_ = 89 ± 1.4 μg/mL Caco2 EC_50_ = 71 ± 2.6 μg/mL THP-1 EC_50_ = 111 ± 3.6 μg/mL A375 EC_50_ = 9.1 ± 0.6 μg/mL	[23]
Rutaceae	*Citrus medica*	Essential oil	Limonene (64.35%)	−	hepatocellular carcinoma HepG2 breast adenocarcinoma MCF-7 colon adenocarcinoma Caco2 Leukemic monocytes THP-1 malignant melanoma A375 immortal keratinocytes HaCat	0.00063–0.9 mg/mL	HepG2: EC_50_ = 0.091 ± 0.012 mg/mL MCF-7: EC_50_ = 0.16 ± 0.012 mg/mL Caco2:EC_50_ = 0.013 ± 0.00001 mg/mL A375:EC_50_ = 0.0057 ± 0.0019 mg/mL HaCat:EC_50_ = 0.024 ± 0.0014 mg/mL	[24]
	*Fortunella margarita*	Essential oil	Limonene (93.78%)	Corfu Island	hepatocellular carcinoma HepG2 breast adenocarcinoma MCF-7 colon adenocarcinoma Caco2 leukemic monocytes THP-1	0.0006–0.86 mg/mL	HepG2 EC_50_ = *n.d.* MCF-7 EC_50_ = *n.d.* Caco2 EC_50_ = 0.1 ± 0.027 mg/mL THP-1 EC_50_ = 0.1 ± 0.0023 mg/mL	[25]
Apiaceae	*Pimpinella anisum*	Essential oil	*trans*-Anethole (88.13%)	−		0.00068–0.97 mg/mL	HepG2 EC_50_ = 0.39 ± 0.0282 mg/mL MCF-7 EC_50_ = 0.3 ± 0.01 mg/mL Caco2 EC_50_ = 0.25 ± 0.04 mg/mL THP-1 EC_50_ =0.11 ± 0.0001 mg/mL	
Lamiaceae	*Mentha spicata*	Essential oil	Carvone (85.41%)	−		0.00067–0.96 mg/mL	HepG2 EC_50_ = 0.22 ± 0.038 mg/mL MCF-7 EC_50_ = 0.284 ± 0.02 mg/mL Caco2 EC_50_ = 0.162 ± 0.0035 mg/mL THP-1 EC_50_ = 0.71 ± 0.004 mg/mL	
	*Ocimum basilicum*	Essential oil	Methyl chavicol (74.92%) Linalool (18.4)	−		0.00068–0.98 mg/mL	HepG2 EC_50_ = 0.18 ± 0.028 mg/mL MCF-7 EC_50_ = 0.17 ± 0.022 mg/mL Caco2 EC_50_ = 0.071 ± 0.003 mg/mL THP-1 EC_50_ =0.67 ± 0.0021 mg/mL	
		Ethanolic extract	Rosmarinic and caffeic acid	Ioannina	cervix adenocarcinoma HeLa melanoma FemX chronic myelogenous leukaemia K562 ovary adenocarcinoma SKOV3	12.5–200 μg/mL	HeLa: EC_50_ = 164.61 ± 2.58 μg/mL FemX: EC_50_ = 191.36 ± 2.42 μg/mL K562: EC_50_ = 157.03 ± 2.25 μg/mL SKOV3: EC_50_ >200 μg/mL	[26]
		Essential oil	Eugenol, Isoeugenol, Linalool	Donation from greenhouse			HeLa: EC_50_=86.11±0.82 μg/mL FemX: EC_50_=96.72±0.65 μg/mL K562: EC_50_=159.78±1.89 μg/mL SKOV3: EC_50_ >200 μg/mL	
	*Marrubium thessalum* Boiss. & Heldr	Methanolic extract	Mainly Phenylethanoid glycosides and flavonoids	Thessalia	Breast adenocarcinoma MCF-7 cervix adenocarcinoma HeLa Colon carcinoma HCT116 Melanoma FM3 PBMCs	50–750 μg/mL	MCF-7: EC_50_ = 417.53 ± 53.4 μg/mL HeLa: EC_50_ = 218.07 ± 64.1 μg/mL HCT116:EC_50_ = 474.07 ± 23.14 μg/mL FM3: EC_50_ = 453.15 ± 37.91 μg/mL 62.20% growth inhibition of PBMCs at 300 μg/mL	[27]
	*Satureja parnassica* spp. *parnassica*	Essential oil	Carvacrol (33.72%) Thymol (17.82%) *p*-Cymene (10.32%) γ-Terpinene (15.47%)	Mt Parnon, Peloponnese	Breast adenocarcinoma MCF-7	0.00000025–0.25% *v*/*v*	EC_50_ = 0.08 ± 0.03% *v*/*v*	[28]
	*Satureja thymbra*		Carvacrol (39.1%) Thymol (12.59%) *p-*Cymene (8.83%) γ-Terpinene (10.61%)	Mt Immitos, Attiki			EC_50_ = 0.002 ± 0.00038%	
	*Origanum vulgare* sub*. hirtum*	Essential oil	Carvacrol (79.58%)	Euboea	Larynx carcinoma Hep-2 Cervix adenocarcinoma HeLa African green monkey kidney Vero Rabbit skin RSC	--	Complete cell death in all cell lines at 0.0001% *v*/*v* Reduction of Vero viability at 0.00002% *v*/*v* (EC_50_ = 0.000027% *v*/*v*)	[29]
	*Origanum Onites* L	Essential oil	Carvacrol (48%)	Northern Greece	Hepatocellular carcinoma HepG2 Breast adenocarcinoma MCF-7 Colon adenocarcinoma HT-29 Malignant melanoma A375 Mouse colon carcinoma CT-26	0.0000589–0.842 mg/mL	EC_50_ 48 h HT-29: 58.00 ± 0.70 µg/mL CT26: 71.70 ± 1.20 µg/mL EC_50_ 72 h A375: 8.90 *±* 0.70 µg/mL MCF-7: 10.00 *±* 1.70 µg/mL HepG2: 23.00 ± 4.20 µg/mL HT-29: 0.35 ± 0.20 µg/mL CT26: 1.10 ± 0.30 µg/mL Attenuation of colon cancer cell migration Induction of Apoptosis-Related Morphological Changes	[30]
					Colon cancer mouse model (in vivo)	0.370 g/kg b.w	52% lower mean tumor volume	
Hyperi-caceae	*Origanum dictamnus* (dittany)	Essential oil	Carvacrol (52.18%) γ-terpinene (8.4%)	Crete	Hepatocellular carcinoma HepG2	0.00007–0.1% *v*/*v*	EC_50_ = 0.0069 ± 0.00014% *v*/*v*	[31]
		Essential oil	*p*-cymene (32.7%) γ-terpinene (12.4%)	Crete	Colon carcinoma LoVo Hepatocellular carcinoma HepG2	2.5–100 µg/mL	LoVo: EC_50_ 24 h = 84.76 ± 1.03 μg/mL EC_50_ 48 h = 72.26 ± 1.05 μg/mL HepG2: Reduction of cell viability	[32]
		Dichloromethane residue	Ursolic acid	Crete	Mouse leukemia P-388 Non-small cell lung carcinoma NSCLC-N6	--	P-388: EC_50_ = 8 μg/mL NSCLC-N6: EC_50_ = 14 μg/mL	[33]
		Ethanolic extract	−				Almost inactive	
		Infusion	Carvacrol 3745.3 **μg/cup (200 mL)** Rosmarinic acid 7244	Crete	Colon carcinoma HT-29 Prostate adenocarcinoma PC-3	0.2, 0.6, 1 μg/μL	PC3 antiproliferative activity: *Origanum dictamnus* >marjoram>sage> rosemary>St John’s wort>thyme HT-29 antiproliferative activity: *Origanum dictamnus* >rosemary> thyme>sage>marjoram>St John’s wort IL-8 decrease in HT-29: sage> *Origanum dictamnus* >thyme> marjoram>St John’ s wort> rosemary IL-8 decrease in PC-3: Thyme>sage > *Origanum dictamnus >*marjoram > rosemary> St John’s wort Reduced levels of the p65 NF-kB subunit by St John’s Wort in HT29 and thyme in PC3	[34]
	*Rosmarinus* *officinalis* (rosemary)		Rosmarinic acid 669 Carvacrol 362.3					
	*Thymus* *Vulgaris* (thyme)		Carvacrol 182,138 Thymol 80,655					
	*Origanum* *majorana* (marjoram)		Rosmarinic acid 9674.4 Carvacrol 2796					
	*Salvia* *officinalis* (sage)		Rosmarinic acid 8082.7 Carvacrol 3745					
	*Hypericum* *perforatum* (St John’s Wort)		Epicatechin 29,275.4 Catechin 3448.2 Quercetin 3134	Central Macedonia				
		Methanolic extract	Hypericin hyperforin	Wild collected 38/15, 41/15: North-West Macedonia 43/15: North–Central Macedonia	Colon adenocarcinoma Caco2	0.01–100 μg/mL	38/15 & 41/15: Did not affect cell viability 43/15: Reduced viability to 82.04% only at the highest concentration	[35]
				Cultivated NAT, D-4: North Greece			NAT: Reduced viability to 41.14% only at the highest concentration D-4: Did not affect cell viability	
		Aqueous solution (AS)	−	Ioannina	Urinary bladder carcinoma T24 Rat urinary bladder carcinoma NBT-II	AS and MS: 0.63-20 μL/mL ME: 13-40 μg/mL PEE: 2–12 μg/mL	T24:AS EC_50_ = 4.7 μL/mL MS EC_50_ = 1.1 μL/mL ME EC_50_ = 18 μg/mL PEE EC_50_ = 5 μg/mL NBT-II: ME EC_50_ = 13 μg/mL PEE EC_50_ = 7 μg/mL Indication of apoptosis induction in T24 and NBT-II cells by PEE extract	[36]
		Methanolic solution (MS)	−					
		Methanolic extract (ME)	Hyperforin (7.62%)					
		Petroleum ether extract (PEE)	Hyperforin (18.9%)					
		Methanolic extract	−	Monodendrι, Epirus	Colon carcinoma Caco2 Hepatocellular carcinoma HepG2	10–100 mg/mL	EC_50_ of all extracts against all cell cancer lines > 100 mg/mL, apart from: *H.* *empetrifolium* Wild Parnitha: Caco2 EC_50_ = 51.3 mg/mL HepG2 EC_50_ = 43.7 mg/mL *H.* *empetrifolium* Wild Hymettus: Caco2 EC_50_ = 54.2 mg/mL HepG2 EC_50_ = 45.8 mg/mL *H.* *empetrifolium* Wild Parnon: Caco2 EC_50_ = 29 mg/mL HepG2 EC_50_ = 25.1 mg/mL	[37]
	*Hypericumempetrifolium* Wild		−	Mt. Parnitha, Attiki Mt.Hymettos, Attiki Parnon, Arkadia				
	*Hypericum rumeliacum* Boiss.		−	Mt. Parnassos, Viotia				
	*Hypericum perfoliatum* L.		−	Korinthos Preveza				
	*Hypericum triquetrifolium* Turra		−	Rafina, Attiki Lakonia Crete				
Lamiaceae	*Teucrium brevifolium*	Essential oil	Spathulenol (9%) δ-cadinene (4.2%)	Karpathos Island	Lung carcinoma COR-L23 Colorectal adenocarcinoma Caco2 Amelanotic melanoma C32	5–200 μg/mL	COR-L23: EC_50_ = 80.7 ± 2.1μg/mL Caco2: EC_50_ = 164 ± 2.1 μg/mL C32: EC_50_ >200 μg/mL	[38]
	*Teucrium flavum*	Essential oil	caryophyllene (12.2%) 4-vinyl guaiacol (9.7%) caryophyllene oxide (7.9%)	Pelion mountain			COR-L23: EC_50_ = 104 ± 2.1 μg/mL Caco2: EC_50_ >200 μg/mL C32: EC_50_ >200 μg/mL	
	*Teucrium montbretii* ssp. *heliotropiifolim*	Essential oil	Carvacrol (13.9%) caryophyllene oxide (12.7%)	Karpathos Island			COR-L23: EC_50_ = 143 ± 2.1 μg/mL Caco2: EC_50_ = 92.2 ± 2.1 μg/mL C32: EC_50_ = 135 ± 2.1 μg/mL	
	*Teucrium polium* ssp. *capitatum*	Essential oil	Carvacrol (10.1%), caryophyllene (9.8%) torreyol (7.6%)	Crete			COR-L23: EC_50_ = 104 ± 2.1 μg/mL Caco2: EC_50_ = 52.7 ± 2.1 μg/mL C32: EC_50_ = 91.2 ± 2.1 μg/mL	
Asteraceae	*Matriacaria chamomilla* (chamomile)	infusion	Carvacrol 9321.5 **μg/cup (200 mL)** Chlorogenic acid 4800.34	Crete	Colon carcinoma HT-29 Prostate adenocarcinoma PC-3	0.2, 0.6, 1 μg/μL	HT-29: Pink savory most effective, chamomile least cytotoxic PC-3: Pink savory most effective, chamomile and mountain tea least cytotoxic IL-8 decrease in HT-29: mountain tea > pennyroyal > chamomile > *Origanum microphyllum* > pink savory> *Origanum* vulgare IL-8 decrease in PC-3: *Origanum microphyllum* > pennyroyal > pink savory *> Origanum vulgare* > chamomile > mountain tea Reduction of p65 NF-kB subunit only by chamomile in HT-29 cells	[39]
Lamiaceae	*Origanum microphyllum*		Carvacrol 23,274					
	*Sideritis syriaca* (mountain tea)		Carvacrol 1220 Chlorogenic acid 828.49					
	*Origanum vulgare*		Carvacrol 177,855 Thymol 2545					
	*Satureja thymbra* (pink savory)		Carvacrol 87,814 Thymol 34,382					
	*Mentha pulegium* (pennyroyal)		Carvacrol 3755 Thymol 1727					
			-	Lesvos Island	Breast adenocarcinoma MCF-7 Colon carcinoma Caco2 Hepatocellular carcinoma HepG2 Normal mouse adipose areolar cells	10–100 μg/mL	EC_50_ of all extracts against all cell cancer lines > 75 μg/mL, apart from: *Clinopodium vulgare*: MCF-7 EC_50_ = 60.4 μg/mL *Rosmarinus officinalis*: MCF-7 EC_50_ = 42.6 μg/mL *Thymus parnassicus* Halácsy: Caco2 EC_50_ = 44.6 μg/mL HepG2 EC_50_ = 50.3 μg/mL MCF-7 EC_50_ = 54.7 μg/mL *Rosmarinus officinalis* was the most cytotoxic against a normal mouse adipose arelar cell lines at 100 μg/mL	[40]
	*Micromeria juliana* (L.) Bentham ex Reinchenb.	Methanolic extract	-	Mt Parnassos, Viotia				
	*Nepeta argolica* Bory et Chaub. subsp. *argolica*		-	Mt. Parnitha, Attiki				
	*Phlomis pungens* Willd.		-	Mt Parnassos, Viotia				
	*Rosmarinus officinalis* L.		-	Zante Island				
	*Satureja graeca* L		-	Mt. Penteli, Attiki				
	*Scutellaria rupestris* Boiss. & Heldr		-	Mt. Parnitha, Attiki				
	*Sideritis sipylea* Boiss		-	Lesvos Island				
	*Stachys spruneri* Boiss.		-	Mt. Parnitha, Attiki				
			-	Mt. Pateras, Attiki				
	*Teucrium divaricatum* Heldr. ssp. *divaricatum*		-	Mt.- Pateras, Attiki				
	*Teucrium polium* L.		-	Mt.Hymettus, Attiki				
	*Thymus atticus* Celak.		-	Mt. Parnitha, Attiki				
	*Thymus longicaulis* C. Presl		-	Trikala				
	*Thymus parnassicus* Halacsy		-	Mt. Kitheron, Attiki				
	*Thymus samius* Ronniger & Rech		-	Samos Island				
	*Thymus teucrioides* Boiss. & Spruner		-	Domokos, Fthiotida				
	*Clinopodium vulgare* L		-	Mt. Parnitha, Attiki				
	*Lavandula stoechas* L.		-	Lesvos Island				

*Citrus medica* (citron) is an evergreen shrub or small tree that produces citron, probably the only citrus fruit known in Europe in ancient times that has been extensively used for its medicinal properties as reviewed in detail in [41]. It was found that the oil of citron is cytotoxic against human cancer cell lines in vitro, especially against the colon carcinoma Caco2, while it affected a non-tumorigenic human cell line to a lesser extent. Its cytotoxicity was higher compared to its major component, limonene [24].

*Lippia citriodora* (or *Aloysia citriodora, Aloysia triphylla)*, commonly known as lemon verbena, belongs to the Verbenaceae family. It was cultivated in South and Central America and was brought to Europe in the 17th century [42]. Its leaves are mainly used for the preparation of infusions and decoctions, usually consumed for the relief of gastroinstestinal problems, but they have also been used as anti-spasmodic, diuretic, or sedatives [43]. There are published data on the antiproliferative activity of the essential oil from plants of different origin (Morocco and Colombia), but only one study on the bioactivities of the Greek oil exists. Lemon verbena oil demonstrated high antiproliferative activity against a panel of human cancer cell lines (A375 > Caco2 > HepG2 > MCF-7 > THP-1), which was lower than its major component, citral, possibly due to the lower DNA-damaging effect it was shown to possess [23].

*Satureja parnassica* spp. *parnassica* is an aromatic plant endemic to Central and Southern Greece. Although literature is sparse regarding its activity, existing studies reveal a potent antimicrobial and insecticidal profile of its essential oil [44,45]. *Satureja thymbra* (common names: Thyme-leaved savory, pink savory, thrubi) is an aromatic plant endemic to the Mediterranean region and is the most common specimen of the *Satureja* genus. It is regarded as oil-rich and its essential oil has been tested against several models and was shown to possess potent antioxidant, antifungal, antibacterial, antiviral, and insecticidal properties [44,45,46,47,48,49]. In a study published in 2016, the antiproliferative activity of the essential oils of *S. thymbra* and *S. parnassica* collected after their flowering season (August and September, respectively) and their major components were investigated [28]. It was shown that the oil of *S. thymbra* was more cytotoxic against MCF- 7 cells, possibly due to its higher content in carvacrol, a phenol with significant bioactivities [50].

*Origanum vulgare* spp. *hirtum* (also known as Greek oregano) is considered the best for culinary purposes. It is the most common *O. vulgare* subspecies in the Greek area and has shown great variation in its chemical composition depending on the cultivation area [51]. Its essential oil was studied against two normal cell lines of monkey and rabbit origin (Vero, RSC, respectively) and two human cancer cell lines (Hep-2, HeLa) without exhibiting any cancer-specific cytototoxicity. In fact, the oil was found to be more cytotoxic against Vero cells compared to the other cell lines [29].

Another member of the *Origanum* genus, *Origanum Onites* L., is commercially known as Turkish oregano and can be mainly found in Turkey, but also in Greece and a small part of Sicily. Different extracts of the plant are used for the relief of common health problems and this has led to the investigation of their pharmacological properties, as well (reviewed in [52]). In a recent study, the essential oil of *O. Onites* isolated from plants collected in Northern Greece has shown dose-dependent antiproliferative activity in vitro, especially against the colon cancer cell line HT-29 (followed by A375, MCF-7 and HepG2 carcinomas). Treatment with the oil caused time-dependent cytotoxicity against HT-29 and CT26 cells (murine colon cancer cell line), attenuation of their migration, and apoptosis-like features, while oral administration for 13 days significantly inhibited the growth of CT26 tumors in mice [30].

*Origanum dictamnus* (dittany of Crete), is a perennial plant endemic to the island of Crete that grows at different altitudes. The essential oil of the plant demonstrated time-dependent cytotoxicity against the LoVo colon cancer cell line, while it reduced the viability of HepG2 cells [31,32]. A dichloromethane residue was almost two-fold more cytotoxic against the leukemic cells P-388 compared to the lung carcinoma NSCLC-N6; however, the ethanol extract appeared to be almost inactive against these cell lines. The difference in their activity could be attributed to the presence of ursolic acid in the dichloromethane residue, which was shown to be more cytotoxic than the residue against the cancer cell lines [33]. Finally, the herbal infusion of the aerial parts of the plant was the most cytotoxic against HT-29 and PC-3 cells compared to infusions from other Greek plants [34].

In the same study, *Rosmarinus officinalis* (rosemary), *Thymus vulgaris* (thyme), *Origanum majorana* (marjoram), *Salvia officinalis* (sage), and *Hypericum perforatum* (St John’s Wort) were also studied. The infusions of marjoram and St John’s wort were the least cytotoxic against HT-29 cells, while PC-3 were the least sensitive against thyme. St John’s Wort in HT29 and thyme in PC3 cells caused reduction of the levels of the p65 NF-kB subunit and all infusions decreased the levels of the pro-inflammatory interleukin 8, in an order depending on the cell line. The authors could not make a correlation between the antiproliferative profile of the extracts and their composition.

A similar study was conducted a year before, in 2013, where the antiproliferative activity of herbal infusions from leaves and flowers of the plants *Origanum vulgare, Origanum microphyllum*, *Satureja thymbra* (pink savory), *Sideritis syriaca* (mountain tea), *Mentha pulegium* (pennyroyal), and *Matricaria chamomilla* (chamomile) was tested [39]. The authors showed that pink savory was the most cytotoxic against HT-29 and PC-3 cells, while chamomile infusion and chamomile together with mountain tea were the least effective against HT-29 and PC-3 cells, respectively. However, the infusion of chamomile was the only one that caused statistically significant reduction of the p65 NF-kB subunit in HT-29 cells. In addition, all infusions were able to decrease the levels of interleukin-8 in the following order: HT-29: mountain tea > pennyroyal > chamomile > *Origanum microphyllum* > pink savory> *Origanum* vulgare, PC-3: *Origanum microphyllum* > pennyroyal > pink savory *> Origanum vulgare* > chamomile > mountain tea.

St. John’s Wort is mainly known for its use against depression and anxiety; however, there is emerging evidence for its potential against cancer, too [53]. The aqueous and methanolic solution (AS and MS) as well as the methanolic and petroleum ether extracts (ME and PEE) of St John’s Wort from Epirus were tested against the urinary bladder carcinoma T24 and the rat urinary bladder carcinoma NBT-II. The MS was more potent than the AS against T24 cells and the same applied for the PEE compared to the ME. The PEE was also more cytotoxic than the ME against the NBT-II cell line, which is attributed to yet unidentifiable components of the herb. PEE was also shown to induce apoptosis in both cell lines using the TUNEL assay [36]. A methanolic extract of the plant together with methanolic extracts from four other species of the genus *Hypericum* (*H*. *empetrifolium, perfoliatum, rumeliacum, triquetrifolium*) showed diverse antioxidant activity and cytotoxicity against Caco2 and HepG2 cells, with the extracts of *H. empetrifolium* Wild from three different locations being the most cytotoxic; however, the concentrations used were very high [37]. Methanolic extracts from aerial parts of different St John’s wort’s chemotypes from different locations (cultivated and wild) were tested against Caco2 cells [35]. The highest activity was presented by one of the cultivated chemotypes, followed by one of the wild populations, while the other extracts did not affect cell viability at all. They also included a commercial sample in their analysis, but it was not of Greek origin. The authors found positive correlation between the antiproliferative activity and the content in rutin and hyperforin, two main components of *Hypericum* species. Differences in their content were attributed to different environmental conditions at the growing sites of the plants.

The methanolic extracts of pennyroyal and rosemary and other 16 plants mainly from Attiki region and Lesvos island (*Clinopodium vulgare* L., *Lavandula stoechas* L., *Micromeria juliana* L. Bentham ex Reinchenb., *Nepeta argolica* Bory et Chaub. subsp. *argolica*, *Phlomis pungens* Wild., *Satureja graeca* L., *Scutellaria rupestris* Boiss. & Heldr., *Sideritis sipylea* Boiss., *Stachys spruneri* Boiss., *Teucrium divaricatum* Heldr. subsp. *divaricatum*, *Teucrium polium* L., *Thymus atticus* Čelak., *Thymus longicaulis* C. Presl, *Thymus parnassicus* Halacsy, *Thymus samius* Ronniger & Rech., *Thymus teucrioides* Boiss. & Spruner) have been investigated against three human cancer cells lines (MCF-7, Caco2, HepG2) and normal mouse adipose areolar cells [40]. Rosemary was the most potent against MCF-7 cells, while *Thymus parnassicus* Halácsy against Caco2 and HepG2; however, rosemary extract was also the most cytotoxic towards the normal mouse cell line used. In the same study, the extracts were also tested for their toxicity against brine shrimps and pennyroyal was found to be the most cytotoxic (LC_50_ = 347.3 μg/mL).

The genus *Teucrium* can be found worldwide but mostly in the Mediterranean region. Menichini and her colleagues in 2009 studied the antiproliferative potential of four *Teucrium* species from Greece [38]. More specifically, the essential oil from the aerial parts of *T. brevifolium*, *T. flavum*, *T. montbretii* ssp. *Heliotropiifolium,* and *T. polium* ssp. *capitatum* were isolated and studied against lung carcinoma COR-L23, colorectal adenocarcinoma Caco2, and amelanotic melanoma C32. They observed that *T. brevifolium* oil was the most potent against COR-L23 cells, while *Teucrium polium* ssp. *capitatum* was the most potent against Caco2 and C32 cells. The authors attributed the diverse activity to the different classes of compounds present in the oils (different classes of sesquiterpenes). All oils also exhibited anti-inflammatory activity, being able to inhibit LPS-induced NO production, with *T. brevifolium* possessing the highest capacity.

*Crocus sativus* L. is an aromatic flowering plant, which produces saffron spice, the most expensive spice in the world, known as “red gold” [54]. The herb has been widely used by ancient Indian, Arabic, and Chinese civilizations as medicine, but is also studied nowadays for its therapeutic potential against several pathological conditions including depression, diabetes, and cancer [55]. In a recent study, Giakoumettis and his colleagues investigated the antiproliferative effect of an ethanolic saffron extract against rat glioma cells C6 alone or in combination with the chemotherapeutic drug temozolomide (TMZ). They showed that the saffron extract is cytotoxic against the cell line after 48 h of treatment and in some combinations, it enhances the cytotoxicity of the drug, too. In addition, they demonstrated that the extract does not induce apoptosis, but calpain-dependent death, possibly through the activation of autophagy and also impairs the colony formation potential of the cells [22].

The genus *Marrubium* consists of approximately 50 species native to temperate regions of the world. The methanolic extract of the species *Marrubium thessalum* Boiss. & Heldr from Olympus Mountain was tested against four commonly diagnosed cancer types: Breast adenocarcinoma MCF-7, cervix adenocarcinoma HeLa, colon carcinoma HCT116, and melanoma FM3. It was found to be more cytotoxic against HeLa cells, while exhibiting similar but lower cytotoxic potency in the other cell lines. In addition, the methanolic extract was also tested against human peripheral blood mononuclear cells (PBMCs) where it was found to be the most cytotoxic compared to some of its phenolic compounds (acteoside, leucosceptoside A, forsythoside B, alyssonoside, isoacteoside decaffeoyl-acteoside) indicating that it does not selectively kill cancer cells, in contrast to its components, which are promising agents for further studies [27].

Another member of the Lamiaceae family, the genus *Ocimum* consists of 30 species that are cultivated all around the world. *Ocimum basilicum,* also known as sweet basil, is one of the most popular food spices in the world and has been studied for its antimicrobial and pharmaceutical properties [56]. In 2014, the essential oil and ethanolic extract of sweet basil from Greece were studied for their action against the cervix adenocarcinoma HeLa, melanoma FemX, chronic myelogenous leukemia K562, and ovary adenocarcinoma SKOV3 cells lines [26]. The researchers observed that both extracts showed similar activity against SKOV3 and K562 cells, while the oil was two-fold more cytotoxic against HeLa and FemX cells. The oil exhibited the highest and similar activity against HeLa and FemX cells, followed by K562 and SKOV3. The ethanolic extract, on the other hand, was more cytotoxic against K562 cells, followed by HeLa, FemX, and SKOV3. Their activities were considered “mild but notable” compared to their main components. In general, with the exception of few cases, the components exhibited more significant activity and were considered to be responsible for the activity of the extracts. Those components were caffeic and rosmarinic acids for the ethanolic extract and eugenol and isoeugenol for sweet basil oil. Furthermore, the chemical composition of basil oil together with the essential oils isolated from the plants *Fortunella margarita* (kumquat), *Mentha spicata* (spearmint), and *Pimpinella anisum* (anise) was analyzed and their biological properties (antimicrobial, antioxidant, antiproliferative) were determined [25]. All of these plants are widely used for culinary purposes in Greece and as flavorings in beverages, gums, ice creams, etc. None of the essential oils exhibited significant antioxidant activity in vitro, whereas their cytotoxicity depended on the cell line used (HepG2, MCF-7, Caco2, THP-1). In general, sweet basil oil was the most cytotoxic against most of the cancer preclinical models used in the study, while kumquat and anise oils were the most efficient against THP-1 cells. Spearmint oil demonstrated an intermediate effect and it was the least cytotoxic against THP-1 cells.

## 4. Conclusions

Cancer incidence and mortality are constantly increasing, and novel strategies are required for its prevention and treatment. Products of natural origin have been used for their medicinal properties since ancient times and in the last years interest in these properties has increased. Currently, numerous studies exist investigating their pharmaceutical potential, including their chemopreventive and chemotherapeutic effect. Although the number of aromatic plant extracts of Greek origin that have been studied for their antiproliferative activity is limited, existing data against several cancer types are promising and encourage further exploitation of the country’s unique flora diversity. Undoubtedly, further exploration of the molecular mechanisms of action of the extracts and their components is required not only in vitro but in vivo as well, to verify and establish their activity for their future use in the battle against cancer.
